# What do molecules do when we are not looking? State sequence analysis for stochastic chemical systems

**DOI:** 10.1098/rsif.2012.0633

**Published:** 2012-09-12

**Authors:** Pavel Levin, Jérémie Lefebvre, Theodore J. Perkins

**Affiliations:** 1 Ottawa Hospital Research Institute, 501 Smyth Road, Ottawa, Ontario, Canada, K1H 8L6; 2 Department of Biochemistry, Microbiology and Immunology, University of Ottawa, Ottawa, Ontario, Canada; 3 School of Electrical Engineering and Computer Science, University of Ottawa, Ottawa, Ontario, Canada

**Keywords:** continuous time Markov chains, stochastic chemical kinetics, trajectories, human immunodeficiency virus drug resistance, Efavirenz, ion channels

## Abstract

Many biomolecular systems depend on orderly sequences of chemical transformations or reactions. Yet, the dynamics of single molecules or small-copy-number molecular systems are significantly stochastic. Here, we propose state sequence analysis—a new approach for predicting or visualizing the behaviour of stochastic molecular systems by computing maximum probability state sequences, based on initial conditions or boundary conditions. We demonstrate this approach by analysing the acquisition of drug-resistance mutations in the human immunodeficiency virus genome, which depends on rare events occurring on the time scale of years, and the stochastic opening and closing behaviour of a single sodium ion channel, which occurs on the time scale of milliseconds. In both cases, we find that our approach yields novel insights into the stochastic dynamical behaviour of these systems, including insights that are not correctly reproduced in standard time-discretization approaches to trajectory analysis.

## 1. Introduction

Stochasticity is a well-documented phenomenon in biomolecular systems. Advances in microscopy techniques and elegant experiments have revealed inherent stochasticity in the expression of genes and the localization of their products [1–3]. Indeed, there has been an explosion of work on stochasticity in gene expression over the past decade [4–7]. The signalling pathways that allow cells to sense and react to their environments are also subject to molecular stochasticity. For example, the well-known ‘run and tumble’ behaviour of *Escherichia coli* in response to chemical gradients [8,9] is influenced not only by the external gradient but also by stochastic molecular noise in ligand binding to receptors and in the internal protein interactions that convey the signal to the flagellar motor [10,11]. At an entirely different time scale, we observe stochastic mutations to the DNA, either within an individual or at the species level. Such mutations enable evolution, and thus phylogenetic modelling and inference are often based on probabilistic formalisms [12,13]. In neuroscience, apparently stochastic behaviour is observed at many levels of organization, including the stochastic opening and closing behaviour of single ion channels, which became clear with the advent of single-channel patch clamp current recordings [14,15].

Many stochastic molecular systems are modelled formally using continuous-time Markov chains [16]. Such a chain can exist in a discrete set of possible states. A state may represent a particular conformation of a protein molecule, the set of proteins bound in a complex, the binding state of a gene's promoter, the number of mRNAs or proteins expressed from a certain gene in a particular cell, the mutational state of a single nucleotide in the genome or even the sequence of the entire genome itself. A continuous-time Markov chain transitions randomly through a sequence of different states at random moments in time. At any time, the current state of the system probabilistically influences both how long the system will ‘wait’ before transitioning to a new state, and to which state the system will transition next. Using the continuous-time Markov chain formalism, it is possible to model things such as the relative stability or instability of different molecular states, energetic barriers to different transformations, concentration-dependence of certain reactions and so on. Stochastic chemical kinetic models [17], which are popular in the stochastic gene expression literature, implicitly define continuous-time Markov chains. Typical formulations of stochastic Petri nets do the same [18]. So, either directly or indirectly, the formalism of continuous-time Markov chains underlies much modelling and analysis of stochastic chemical systems.

If we have a continuous-time Markov chain model of a real-world system, then we can use the model to make predictions about the system. For instance, we might take the steady-state probabilities of the chain as predictions of what we would likely see if we were to observe the state of the real system at some arbitrary time. Alternatively, if we knew the state of the real system at some time, then we could use the model to compute the probabilities of different possible states at future times. However, a deeper understanding of the system can be gleaned by analysing its *pathwise* behaviour. For instance, protein folding and protein complex assembly are inherently sequential processes in which each stage sets up the possibilities for the next stage. Similarly, gene regulation can depend not just on the factors present, but also on the order in which they bind. Phenomena such as cooperative binding, DNA-looping and histone modifications make the achievement of a given regulatory state an inherently sequential process.

Traditionally, there are two main approaches to studying the pathwise behaviour of a continuous-time Markov chain, each with its strengths and weaknesses. One approach is to discretize time and to use discrete-time path analysis methods [19]. For instance, once time has been discretized, it is easy to compute the most probable path the system will follow, using dynamic programming. However, there is some arbitrariness in choosing the time step for the discretization, and this choice can influence both one's results and the complexity of the computations. The behaviour in the limit of infinitesimal time step size can be computed efficiently, mollifying these problems [20]. However, as we have argued before [20], and as we explain again in the next section, approaches based on time discretization, even in the infinitesimal limit, often produce implausible or non-representative system paths with poor biological relevance.

The other main approach for studying paths of continuous-time Markov chains is to use stochastic simulation algorithms [17,21,22]. By simulating a large number of random trajectories, one can visualize system paths and estimate various aspects of pathwise behaviour. Moreover, simulation is computationally straightforward and readily parallelized. However, methods based on random simulations are inherently approximate; they do not produce exact answers. The most straightforward simulation methods also deal poorly with low-probability events or situations in which we have boundary conditions to satisfy. For instance, suppose we observe the state of a system at two different times, and we are interested in paths between those two states. We can simulate paths from the initial state, but many or even all of them may not reach the proper final state, so that none of them helps us to understand what the real system might have done between our two observations.

We propose a new approach to studying the pathwise behaviour of continuous-time Markov chains that we dub *state sequence analysis*. Our key innovation is to focus on which sequences of states are likely to occur, and not to concern ourselves with the exact timing with which those states are visited. For instance, if we knew that a protein molecule folded in a certain amount of time, we could ask what folding path it likely took, without worrying about the exact times that it entered each intermediate state. Often, the exact timing of events is not nearly as important as which events occur, hence our focus on the state sequence. It turns out that focusing on the state sequence also avoids some of the difficulties involved in simulation-based and time-discretization approaches mentioned in the previous paragraphs. Those methods, it must be pointed out, do not focus on the state sequence alone. For instance, when we simulate a random trajectory, we generate not just a sequence of states, but also the exact amounts of time spent in each state. Similarly, if we discretize time and compute a maximum-likelihood path, that path specifies the state of the system at every time step—again, both a sequence of states and the exact amounts of time spent in each one. In our approach, a state sequence implicitly represents a whole family of such system trajectories, but ones that differ only in how much time they spend in each state. In §2.1, we define more carefully what the probability of a state sequence means and how to find the most probable state sequence, given initial and/or final states. In §2.2, we contrast our approach with the more traditional approaches mentioned earlier. We then demonstrate the value of state sequence analysis as an investigative tool by analysing the evolution of drug resistance in human immunodeficiency virus (HIV) (§2.3) and stochastic ion channel dynamics (§2.4). In both domains, we find that state sequence analysis provides novel insights into the dynamics of these systems, which are not captured by previous analysis approaches. Software implementing our approach in both Matlab and R, and scripts that analyse all examples in the paper, can be found at www.perkinslab.ca.

## 2. Results

### 2.1. State sequence analysis for continuous-time Markov chains

A continuous-time Markov chain is a discrete-state continuous-time stochastic dynamical system [16]. We restrict attention to finite-state chains. Thus, at every real-valued time 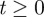, the chain is in some state *X*(*t*) from a finite set of possible states 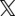. The dynamics of the chain work as follows. Suppose at time *t* the system is in state *X*. A dwell time parameter 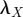 controls how long the system will stay in that state. If 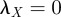, then *X* is called a terminal or absorbing state. The system never leaves such a state. If 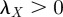, then *X* is non-terminal or non-absorbing. The system will stay in state *X* for a random amount of time that is exponentially distributed with parameter 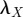 (thus, mean time 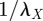), and then transition to a new state 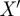 with probability 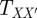. A state can never transition to itself; so 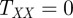 for all 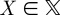. Together, the dwell time parameters *λ* and transition probabilities *T* fully define the dynamics of the chain.

**Figure 1.**
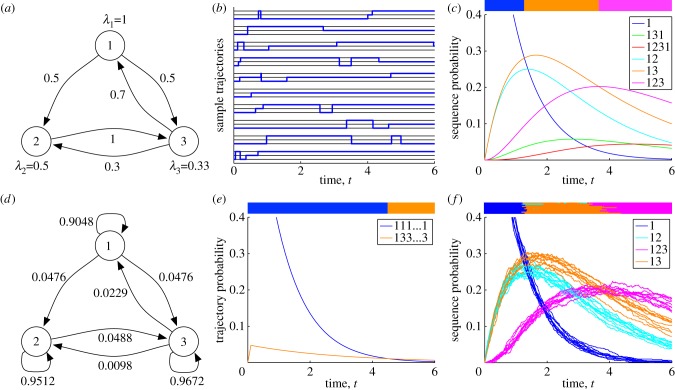
Alternative approaches to analysing probable pathwise behaviours of continuous-time Markov chains. (*a*) An example chain with three states. Dwell time parameters and transition probabilities are shown. (*b*) Ten randomly sampled trajectories of the system, starting from state 1, for a period of 6 s. Each trajectory comprises a specific sequence of states and dwell times. (*c*) The probabilities that the system transits different sequences of states as a function of time, after averaging over the possible transition times. The coloured bar at the top indicates the single most probable state sequence as a function of time, obtained using state sequence analysis. (*d*) A discrete-time Markov chain approximating the continuous-time chain. (*e*) Maximum probability trajectories for the time-discretized chain, obtained using standard dynamic programming techniques. Results differ from (*c*) because (*e*) represents single trajectories, whereas the sequences analysed in (*c*) represent integration over all possible trajectories having the same state sequence. (*f*) Stochastic simulation can be used to estimate state sequence probabilities, but there are uncertainties in the estimates, which sometimes results in incorrect identification of the maximally probable sequence.

A trajectory of a continuous-time Markov chain is a random realization of its dynamics, either for all time 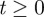 or over some interval of time. Figure 1*b* shows 10 random trajectories generated from the chain specified in figure 1*a*, all starting from state 1. For instance, the first trajectory (the top-most) shows the system residing in state 1 for a little less than 1 s, briefly visiting state 3, returning to state 1 for about 3 s, briefly visiting state 2, and then transitioning to state 3, where it stays until the end of the simulation at *t* = 6 s. It so happens that the last trajectory in figure 1*b* (the bottom-most) visits the exact same sequence of states, (1, 3, 1, 2, 3), although it visits each for a different amount of time. Other random trajectories visit states in different orders. Intuitively, different sequences of states occur with different probabilities. The trajectories shown in figure 1*b* suggest another important point—that the probability of the chain visiting a sequence of states depends on the duration of the trajectory. For instance, if we imagine cutting off the trajectories at *t* = 0.5 s, about half of them will not have even left the initial state. However, by time *t* = 6 s, all trajectories have left state 1 at least once. The fundamental idea of state sequence analysis is that, over a given period of time, different state sequences will occur with different probabilities, and that the most probable sequence provides an important and useful characterization of the dynamics of the system.

Consider an arbitrary state sequence *S* = (*X*_0_, *X*_1_, …, *X*_*N*_). Let us assume that the chain starts in state *X*_0_ at time zero. What is the probability that the chain traverses precisely the sequence of states *S*, and no additional states, in time *t*? In other words, if the system randomly follows one of its infinitely many possible trajectories, what is the chance that the trajectory transits the sequence of states *S* during the first *t* amount of time? This event requires three things to happen: (i) each time the system transitions to a new state, the next state must be the one specified by the sequence *S*, (ii) the system must arrive to the final state *X*_*N*_ before time *t*, and (iii) the system must not leave state *X*_*N*_ until some time after *t*. If we let 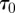, …, 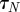 be the random amounts of time the system spends in states *X*_0_, … , *X*_*N*_, then the time-dependent probability of seeing the state sequence *S* can be written as
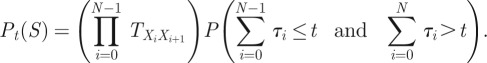
The first term accounts for condition (i) and the second term accounts for conditions (ii) and (iii). Figure 1*c* shows these time-dependent probabilities for several possible state sequences, based on the chain in figure 1*a*. For example, the dark blue curve shows that the probability of the trivial state sequence *S* = (1) decreases exponentially. Indeed, it is equal to precisely 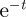, which is just the probability of the system remaining in the initial state *X*_0_ = 1 until at least time *t*. The orange curve shows the probability of the state sequence *S* = (1,3), meaning that the system transitions to state 3 sometime before time *t*, and remains in state 3 until at least time *t*. This probability is zero at *t* = 0 s, peaks at around *t* = 1.65 s, and then slowly declines. (The exact probability is 
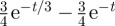
.) For all the state sequences shown except *S* = (1), the probabilities start at zero, increase up to some time and then fall again towards zero. The exact shapes of these curves depend on the dwell time parameters of the states in the sequence and the transition probabilities between them. Intuitively, their unimodal shape represents a trade-off between the two time-dependent conditions, (ii) and (iii). With increasing time, it is more likely that the system has time to visit all the states in the sequence (thus, 
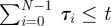
); however, it is decreasingly likely that the system will not have gone on to visit other states as well (invalidating 
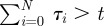
). These opposing influences on the state sequence probability result in the unimodal shape of the curve as a function of time.

In §4.1, we explain in detail how curves such as those in figure 1*c* can be computed. Briefly, for the one-state sequence *S* = (*X*_0_), assuming the chain starts in state *X*_0_, the probability is simply 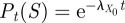. For a longer state sequence 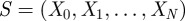, and letting 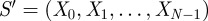 be the one-step-shorter sequence, the probability obeys the differential equation2.1
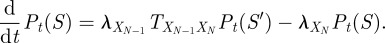
Intuitively, the probability of the state sequence *S* increases to the extent that the shorter sequence, *S*′, is probable and a transition from 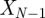 to *X*_*N*_ is likely. The probability of *S* decreases to the extent that a transition out of *X*_*N*_ is likely. The curve for 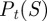 can thus be computed by recursively solving a system of *N* linear differential equations, which can be done by various analytical or numerical means.

The core idea of state sequence analysis is to compute the most probable state sequence that a continuous-time Markov chain follows, given an initial state *X*_0_ and allowing for total time 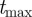. Because there can be infinitely many possible state sequences, it is impossible to simply evaluate the probability of each one. Therefore, the search for a maximum probability state sequence must be somehow limited. Our approach is described fully in §4.2, but we highlight the key ideas here.

Suppose that *S* and *S*′ are two state sequences with the same starting and ending states, 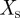 and 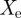. Further, suppose that 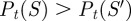 for all 
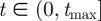
. In other words, for the time period of interest, *S* is a strictly more probable way for the chain to go from 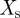 to 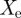 than *S*′ is. In this case, we say that *S* dominates *S*′. When this happens, neither *S*′ nor any single- or multiple-state extension of *S*′ can be part of a maximum probability state sequence, and so can be ignored. (See proofs in §§4.2 and 4.3.) Thus, we propose to find maximum probability state sequences by starting to enumerate all sequences from shorter to longer. However, any time we find a sequence that is dominated by one we have already enumerated, or that dominates any one we have already enumerated, the dominated sequence and any extension of it are discarded. The state sequences whose probability curves are shown in figure 1*c* are precisely those that remain after the enumeration—all other possible sequences are dominated by at least one of these, and are discarded. We prove in §§4.2–4.4 that this approach finds all maximum probability state sequences over the time interval 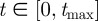. For example, in figure 1*c*, one can easily read off the most probable state sequence for any point in time based on the non-dominated probability curves. Up until about 1.27 s, it is the trivial state sequence (1). From 1.27 to 3.66 s, it is the sequence (1,3), and from 3.66 s until the final time of 6 s, it is the sequence (1,2,3).

Several generalizations of the core state sequence analysis problem are natural. For instance, we may want to restrict attention to a specified final state at time 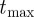. We may want to impose initial and/or final probability distributions over the possible states. We may even be interested in the *K* most probable sequences, instead of the single most probable sequence. These can all be solved as straightforward extensions of our core algorithm, as we describe in §4.2.

### 2.2. State sequence analysis differs from traditional approaches based on time discretization or stochastic simulation

As mentioned in §1, a more traditional approach to path analysis for continuous-time Markov chains is to discretize time and to use dynamic programming procedures to compute maximum probability paths. Let us illustrate this approach, and how it differs from state sequence analysis, for the chain depicted in figure 1*a*. Suppose we discretize time to a resolution of *Δt* = 0.1 s. Then, the continuous-time chain of figure 1*a* is well approximated by the discrete-time Markov chain shown in figure 1*d*. This chain has the same set of possible states as the continuous-time chain, and it has transition probabilities between states, but no waiting time parameters. However, it is allowed for a state to ‘transition’ to itself, and this reflects the tendency of the continuous-time chain to remain in the same state. For example, letting *T*′ denote the transition probabilities of the discrete-time chain, 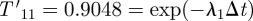, which is the probability that the continuous-time chain does not leave state 1 during *Δt* time. Transitions to a different state also reflect the probability of the continuous-time chain making that transition. For example, 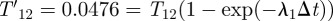, which is the probability that the continuous-time chain would leave state 1 in *Δt* time, and would transition next to state 2.^1^

A trajectory of a discrete-time Markov chain is a random sequence of states generated according to the transition probabilities *T*′. Assuming the chain starts in a given state *X*_0_, the probability of an *N*-step trajectory 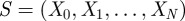 is just 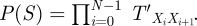 For discrete-time chains, maximum likelihood trajectory inference is well understood. Given the initial state, *X*_0_, a simple dynamic program can compute the single most probable trajectory to any future state *X* at any future time 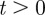.^2^ We computed the most probable trajectories of the discrete-time chain from an initial state of *X*_0_ = 1, allowing for up to 60 transitions, or 6 s. The results are shown in figure 1*e*. Up until 4.6 s, the most probable trajectory is that the system stays in state 1. The probability of this trajectory, as a function of the number of time steps *n*, is 0.9048^*n*^, an exponential decay very similar to the curve seen in figure 1*c*. For times 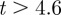 s, however, the single most probable trajectory of the discrete-time chain is (1, 3, 3, …, 3). That is, the chain transitions to state 3 immediately after time step 1, and then keeps following the self-loop on state 3, thus staying there for the rest of the time available. The probability of this trajectory, for number of steps 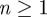, is 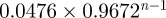. For sufficiently large *n*, this is more probable than the trajectory of staying in state 1. Note, however, that while the non-repeating sequence of states visited by this second trajectory is (1,3), it does not represent all possible trajectories demonstrating the same state sequence. In particular, the probability calculated by the dynamic program (and the Viterbi algorithm would do the same) does not include the trajectory (1, 1, 3, 3, …, 3) or (1, 1, 1, 3, 3, …, 3) and so on. In this sense, the state sequence analysis we propose for continuous-time chains is crucially different from what one obtains from trajectory analysis of a corresponding discrete-time chain. In state sequence analysis, all trajectories that visit the same sequence of states are lumped together, whereas algorithms such as Viterbi count them all separately. In applications to the evolution of drug resistance to HIV (§2.3) and ion channel dynamics (§2.4), we show that this distinction is crucial to obtaining biologically meaningful results.

Another possible approach to path analysis of continuous-time Markov chains is to retain the continuous-time dynamics, and to accumulate statistics based on stochastic simulations [21,22]. For the problem of identifying the most probable state sequence, one could simulate a large number of random trajectories for 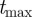 time, and simply check which state sequence occurs most often [23]. We tested this approach on the chain in figure 1*a*, simulating 1000 random trajectories from initial state *X*_0_ = 1 for 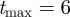 s total time. Then, for each of a discrete set of times 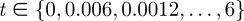 s, we determined: which state sequences had occurred up to time *t* among the 1000 trajectories, the empirical probabilities of these state sequences and the single state sequence with the largest empirical probability. We repeated this entire procedure 10 times, to assess variability in the results, which are shown in figure 1*f*. We found that across the 10 independent replicates of the experiment, four state sequences were estimated to be most probable at different times: (1), (1, 2), (1, 2, 3) and (1, 3). As shown in figure 1*c*, the sequence (1, 2) is not, in truth, maximally probable at any time. Although comparison of figure 1*c*,*f* shows that the approximated probability curves are similar to the exact curves, variability in the simulation-based estimates is large enough in some cases to result in an incorrect assessment of which state sequence is most probable. There is also considerable uncertainty in the times at which different state sequences are most probable. Naturally, sampling more random trajectories would result in more accurate results. We arrived at using 1000 trajectories after finding that 100 trajectories resulted in very noisy probability estimates (data not shown). Although stochastic simulation has the benefit of algorithmic simplicity, its downsides include uncertainty in its results and, as we show in our ion channel analysis, a difficulty in handling boundary value problems.

### 2.3. Acquisition of Efavirenz resistance in HIV

One of the challenges in treating HIV is the development of drug resistance. Recent years have seen the spread of drug-resistant strains among the population [24,25], making treatment more difficult. Even within a single patient, however, the virus frequently mutates and may acquire resistance to the patient's therapy. Mutations in the HIV genome that confer resistance to different therapies have been identified [26–29], and a number of studies have looked at predicting levels of resistance based on the mutational profile of particular strains [30–33].

Comparatively few studies have looked at the dynamics of acquisition of these mutations, including questions such as: Which mutations tend to occur first? Do some mutations facilitate other mutations? Which sequences of mutations are most likely to occur? We demonstrate the use of state sequence analysis in answering such questions, basing our analysis on a dataset previously assembled to address this issue [34]. The data comprise genotypes of the HIV strains in 122 patients on Efavirenz combination therapy for which HIV genotyping was performed at multiple time points. Efavirenz is a potent non-nucleoside reverse transcriptase inhibitor, and a common component of the anti-retroviral drug cocktails used to treat HIV [35]. Yet, acquisition of resistance to Efavirenz is a well-documented problem [36,37]. In the initial analysis of the Bacheler *et al.* data [34], the authors confirmed the presence of a number of previously known resistance-associated mutations. They found that one mutation in particular, K103N, occurred especially frequently. (K103N means that amino acid 103 of the reverse transcriptase gene, normally K, is replaced by N.) Other mutations, such as V108I and P225H, tended to occur later on, and never or almost never without the K103N mutation. In general, patients tended to accumulate more resistance-associated mutation as time went on. However, the detailed dynamics of this process were not studied. Foulkes & De Gruttola [38] used the same data to develop a continuous-time Markov chain model of mutation dynamics. They used a clustering approach to define different mutational ‘states’, reiterating the observations that some mutations were correlated and that patients generally acquired more mutations over time—though they also found that reversion to a state with fewer resistance-associated mutations was possible. Beerenwinkel & Drton [39] analysed specific sequences of acquisition of resistance-mutations using mutagenic tree models. Mutagenic trees model the most probable mutations to occur next, given the sequence of mutations that have already occurred. Thus, there is an explicit assumption that mutations can only accumulate, and never revert. They also associated mutation rates to each tree branch, implicitly defining a continuous-time Markov chain. Their focus on mutational pathways is very similar to what we present below except that, following Foulkes & De Gruttola, we will not assume that mutations are irreversible. Buendia *et al.* [40] also modelled mutational dynamics as a continuous-time Markov chain, with the twist that phylogenetic analysis was used to estimate relationships between different viral copies within each patient, which in turn has some effect on estimated transition probabilities and rates of the chain.

**Figure 2.**
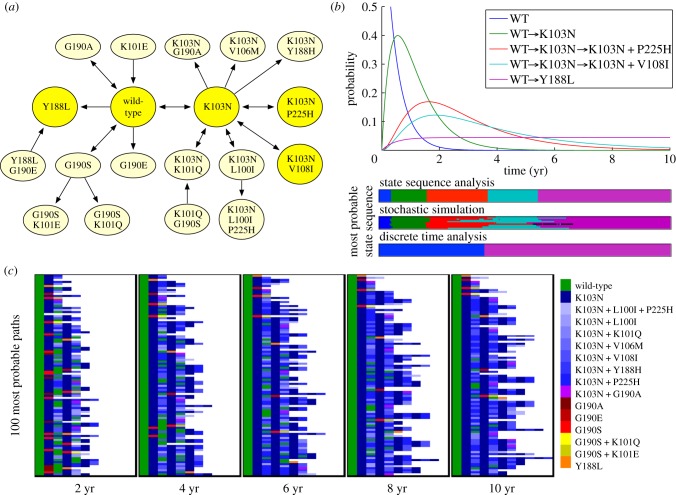
Analysis of within-patient mutational dynamics of HIV subject to Efavirenz combination therapy. (*a*) State transition diagram of the model, estimated based on time-series HIV genotype observations [34]. States are labelled with mutations they include. Details of the estimation method and all fitted model parameters can be found in §4.5. States participating in maximum probability state sequences are highlighted in yellow. (*b*) Probabilities of the most probable state sequences, starting from wild-type, as a function of time during the first 10 years of therapy. Coloured bars along the bottom show the time intervals during which different state sequences (indicated by colour) are maximally probable, according to state sequence analysis, stochastic simulation and time-discretized dynamic programming. (*c*) Depiction of the 100 most probable state sequences at different times. Each row corresponds to a different path, with the sequence of coloured rectangles depicting different states according to the legend at the right. The most probable path is on the top row, and the 100th most probable path is on the bottom row.

To perform state sequence analysis of the dynamics of Efavirenz-resistance mutations, we first had to estimate a continuous-time Markov chain model of the process. We focused on reverse transcriptase mutations highlighted in previous analyses of the Bacheler *et al.* data [36,40] or currently identified as ‘key mutations’ on the Stanford HIV Drug Resistance Database [41]: L100I, K101Q, K101E, K103N, V106M, Y188L, Y188H, G190S, G190E, G190A and P225H. Including the wild-type values, this allows for 4 × 3^2^ × 2^4^ = 576 different possible mutational states. However, only 19 of these occurred in the dataset. These states, and the observed transitions among them, are shown in figure 2*a*. We computed maximum-likelihood estimates of dwell time parameters and transition probabilities following Foulkes & De Gruttola [38,42]. The exact method and resulting parameters can be found in §4.5.

On the basis of the model, we computed the most probable mutation sequences, starting from wild-type, over a 10 year period. This yields a map of the single most probable progressions that a patient similar to those in the study could be expected to experience (figure 2*b*). Unsurprisingly, over a small time period, a patient's HIV is not expected to acquire any mutations. However, after approximately six months, it becomes more probable that the patient's HIV will have acquired mutation K103N. In an array of studies, this is the single most common mutation found in HIV strains with Efavirenz resistance, conferring an approximately 25-fold reduction in efficacy [29,43,44]. Just short of two years, the most probable path in our model adds the mutation P225H, an ‘accessory’ mutation that further reduces susceptibility to Efavirenz [44]. From roughly four years on, an alternative path is most probable—the K103N mutation followed by V108I, which is another exacerbating accessory mutation. Both accessory mutations can subsequently be lost and regained, although such paths are never more probable than other, simpler mutation sequences. From approximately 5.5 years onwards, the single most probable path is to acquire the single mutation Y188L—which confers very strong resistance to Efavirenz [41]. In the dataset, no patient acquiring this mutation ever lost it or acquired other mutations, making the corresponding state in our model an absorbing one.

At the bottom of figure 2*b*, we compare the results of state sequence analysis with maximum-probability path estimation by stochastic simulation and by discrete-time dynamic programming. For the stochastic simulations, we performed 10 independent sets of 1000 simulated trajectories. In nine out of 10 runs, the correct, maximum-probability paths were identified, although there was considerable uncertainty regarding the times at which they are maximally probable. One run incorrectly identified the sequence WT 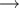 K103N 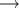 K103N + P225H 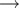 K103N 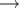 K103N + P225H as maximally probable at around *t* = 6 years. These difficulties with the stochastic simulation approach are analogous to what we saw in the example of §2.2. Also in analogy to that example, the standard time-discretization approach yields much less informative and less realistic solutions. For instance, if we discretize time to a step-size of one day and compute maximum probability trajectories (see [19] or §4.7), we find that up until roughly 3.7 years, the most probable trajectory is that the patient remains with wild-type HIV. The reason for this is essentially that the self-loop probability on the wild-type state is very high (near one), and so it is very difficult for a trajectory to leave the wild-type state. For times *t* larger than 3.7 years, the most probable trajectory is that the patient had developed the Y188L mutation immediately after the start of treatment (on day one) and remained with that mutation until observed at time *t*. Although the transition from wild-type to Y188L is a low-probability event, Y188L is an absorbing state. Its self-loop probability is precisely one, and so for sufficiently large times *t*, the probability of this trajectory is higher than the self-looping trajectory that stays in the wild-type state. Neither trajectory produced by the time-discretized analysis shows any role for the many other mutations that may occur during this time. In comparison, state sequence analysis reveals aspects of the pathwise behaviour far more consistent with the known mutational dynamics.

From years 2 or 3 onwards, the maximally probable mutation sequences shown in figure 2*b* account for a small fraction of the total probability mass. As with all maximum-likelihood approaches, one must consider carefully how representative that maximum is. Looking at the 100 most probable sequences reveals other possible behaviours (figure 2*c*). Many paths are seen to obtain the K103N mutation (dark blue) and then repeatedly add and lose various accessory mutations (lighter shades of blue). These accessory mutations never occur alone; they are always preceded by the K103N mutation—as one can also see directly from the diagram of possible state transitions in figure 2*a*. Sometimes the HIV even returns to the wild-type state, after having acquired drug-resistance mutations. Observations such as these can be important for reconstructing transmission events between individuals; the fact that an individual does not have a mutated strain now does not mean the individual's strain was free of that mutation at an earlier time. The figure also shows that the G190 family of mutations is frequent among the 100 most probable paths, although decreasingly so as time goes on. Paths leading to the Y188L and G190E mutations tend to climb higher in the ranks over time. Because these are absorbing states, the probabilities of paths leading to them asymptote to non-zero values at large times *t*. By contrast, any path leading to a non-terminal state will become increasingly improbable with increasing time, and will be ‘replaced’ with a higher probability, longer path. Indeed, the figure shows the general trend towards these two types of paths as time goes on—paths ending at terminal states, and increasingly long paths jumping among non-terminal states.

### 2.4. Ion channel dynamics

**Figure 3.**
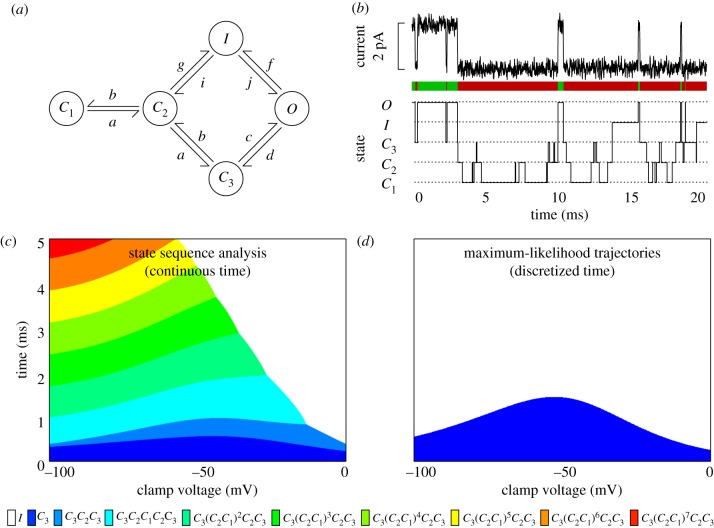
Analysis of the dynamics of a single neural sodium channel. (*a*) Diagram of the model proposed in Vandenberg & Bezanilla [53], describing the gating dynamics of a single sodium channel in the squid giant axon. Transition rates *a*, *b*, *c*, *d*, *f*, *g*, *i* and *j* depend on the voltage of the clamp, as detailed in Vandenberg & Bezanilla [53] (see also §4.6). (*b*) Illustration of the relationship between current recordings of a single ion channel maintained at constant voltage via patch clamp (top), and an associated state trajectory simulated from the model in (*a*). Green and red coloured bars indicate open and closed periods, respectively. (*c*) Maximum probability sequences of closed states depending on the patch clamp voltage and the duration that the channel remains closed, obtained via state sequence analysis. Colour key to state sequences is below the panel. (*d*) Maximum probability sequences of closed states obtained by a time-discretization and dynamic programming. Colour key is below the panel.

Systems operating over much smaller time scales than mutations to the HIV genome may also be investigated using state sequence analysis. Ion channels are proteins regulating the displacement of ions through cellular membranes. They constitute the biophysical basis of cellular excitability that generates action potentials in neurons and contractions in the heart [45–48]. Current recordings from single ion channels demonstrate that they open and close in an essentially stochastic fashion, driven by thermal fluctuations [49–52]. However, the statistics of the durations of open and closed intervals indicate that most channels possess multiple closed states—multiple configurations of the protein that do not allow ions to pass. For example, the seminal modelling work of Vandenberg & Bezanilla [53] showed that the dynamics of sodium channels in squid giant axons may be described by the five-state model shown in figure 3*a*. It has one open state and four closed states, one of which, the inactive or *I* state, has an especially long latency. Moreover, Vandenberg & Bezanilla estimated voltage-dependent transition rates between those states—equivalently, voltage-dependent dwell time parameters for each state and transition probabilities between states.

Although a patch clamp recording reveals whether a channel is open or closed at any time, it does not tell us precisely which closed state the channel is in when it is not passing current (figure 3*b*). Thus, although the overall open/closed state of the channel is experimentally accessible, the exact state of the channel is not. However, the duration of a closed interval has some bearing on the likely state or states of the channel. Focusing on the Vandenberg & Bezanilla model, we applied state sequence analysis to compute the most probable sequences of closed states underlying closed intervals of duration *t* between 0 and 5 ms, for clamp voltages ranging from −100 to 0 mV. We expected that longer closed intervals would be associated with the inactive state *I*, whereas shorter closed intervals would be associated with the more transient *C*_1_, *C*_2_ and *C*_3_ states. In part, this is true, but the answer is highly dependent on voltage, as shown in figure 3*c*. For moderately negative voltages, short sojourns among the closed states are most probably the result of a visit to *C*_3_ and possibly *C*_2_, whereas longer closed periods are most probably the result of a stochastic switch to the inactive state. Contrary to our expectations, however, for highly negative clamp voltages, the most probable explanations for closed periods of the same length involve oscillations between the *C*_1_ and *C*_2_ states, sandwiched between *C*_3_. Thus, for example, if we see the channel stay closed for 5 ms, at 0 mV, this probably results from the inactive state, whereas at −100 mV it does not. Overall, the diagram shows that the behaviour of the ion channel, even while remaining among the closed states, is highly dynamic, and our estimate of its most probable behaviour depends strongly on both the polarization of the membrane and the duration of time for which it is observed to be closed.

Analysing state sequences by stochastic simulation is awkward for the ion channel model. If we performed stochastic simulations, every trajectory would spend a different amount of time among the closed states. Moreover, if we were interested in a specific amount of closed time, 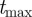, the probability of generating even a single trajectory that stays closed for exactly 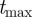 time is zero. Thus, the naive approach described earlier would be useless. The approach could be altered in several ways to account for the final-time constraint of re-entering state *O* at time 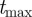. One possibility is that a trajectory reweighting scheme might also be developed [54–57]. We leave the investigation of such alternatives for future work.

The discrete-time dynamic programming approach applies readily. As in the HIV example, however, this approach does not produce meaningful results. Figure 3*d* shows the most probable paths as computed by a time-discretization approach with a time step of 
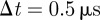
, which results in 10 000 time steps over the 5 ms total time interval. The map shows that short sojourns among the closed states are explained by a single visit to closed state *C*_3_. Longer sojourns are explained by a single visit to the inactive state *I*. Under no conditions does the maximum probability sequence exhibit transitions between different closed states. This is inconsistent with our understanding of these ion channels, and again emphasizes the importance and suitability of state sequence analysis for analysing the probable dynamics of continuous-time Markov chains.

## 3. Discussion

In this article, we proposed state sequence analysis, an approach to investigating stochastic continuous-time discrete-state systems by computing maximum probability state sequences. The probability that the system follows a particular state sequence depends on the transition probabilities between the states, but also on time. Longer state sequences generally require more time to occur, though the dwell time parameters of the states determine the precise dependence. Maximum probability state sequences can be computed efficiently, and may constitute predictions for the future of a stochastic system, estimations of its past behaviour based on limited observations or a means to visualize dynamics in a simpler form.

We demonstrated our approach on two example domains: predicting the acquisition of drug-resistance mutations in HIV patients and estimating the hidden behaviour of ion channels. In the HIV domain, we found that several different mutation sequences were maximally probable, depending on how long the patient has been on Efavirenz combination therapy. When Efavirenz therapy fails in HIV patients, doctors change them to alternative therapies. Knowing the probable cause of that failure, in terms of mutations that may have accumulated in the patient's virus, may be useful information for choosing which alternative therapy to try next. Conversely, knowing the time scales at which different drug-resistance mutations are likely to develop may be useful information when scheduling a patient for periodic follow-up. In analysing the 100 most probable state sequences, we found that repeated gain and loss of mutations, particularly the accessory or ‘secondary’ mutations, is a common phenomenon. We must mention the caveat that the dataset on which we based our study is approximately 10 years old. While Efavirenz remains a standard first-line therapy, changes in drugs that are used in combination with Efavirenz suppress the virus more strongly. This may have altered the selective pressure on the virus, and thus the dynamics with which mutations are accumulated. Additionally, opinion changes over time on exactly which mutations are most important for drug resistance, and why (see the Stanford HIV Drug Resistance Database (http://hivdb.stanford.edu/) [41] for the latest data and opinions on these matters). Nevertheless, our analysis demonstrates that one can take data on mutation dynamics and produce predictions of the most probable sequences of mutational events leading to drug resistance, and the timing with which they are likely to occur.

In the sodium ion channel domain, we showed that our technique can be used to ‘look inside’ time intervals when a patch-clamp recording tells us only that the channel is closed. The behaviour of the channel during this time has long been experimentally inaccessible, and so any predictions or information that one can deduce about the behaviour of the channel during this time are valuable. In particular, we showed that the most likely behaviour of the channel is strongly dependent on both the observed duration of the closed interval and the patch clamp voltage. For mild clamp voltages, short closed intervals are attributed to transient visits to the *C*_3_ closed state, whereas long closed intervals are attributed to the channel getting ‘stuck’ in the high-latency inactive state *I*. However, for strongly negative patch clamp voltages, such as −100 mV, we find it is more probable that the chain oscillates between the *C*_1_ and *C*_2_ closed states, before returning to the open state. In both the HIV and ion channel examples, we found that retaining the continuous-time dynamics of these discrete-state systems, as opposed to discretizing time, was essential to reproducing biologically plausible and meaningful predictions of pathwise behaviour.

We do not yet have a general result on the computational complexity of state sequence analysis. The time the algorithm takes depends on how successfully the dominance criterion is able to prune the otherwise exhaustive search through the set of possible state sequences. The number of non-dominated sequences depends in a complex way on the transition probabilities and the dwell time parameters; so this is difficult to assess in general. In the special case that all dwell time parameters are equal to a common value *λ*, then the probability of any state sequence 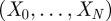 is just 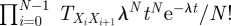 Thus, between any two states, there is at most one dominant sequence of each length (ignoring ties). Assuming a unique start state, no more than 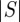 possible extensions to each sequence, and assuming we need not examine sequences longer than 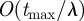 steps, the total number of sequences that need to be considered is 
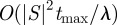
—a time complexity that is polynomial in the state set size and pseudopolynomial in the final time, just as for discrete-time dynamic programming approaches [19]. In practical terms, our Matlab implementation of state sequence analysis, running on a 2 GHz Intel i7 MacBook Pro, took 4.6 s to analyse the example in §2.1 using a numerical grid of 10^4^ points to represent the time interval. By comparison, simulating one set of 1000 stochastic trajectories took just 0.23 s. However, analysing those trajectories on a 10^4^ point numerical grid to identify all the different state sequences that occurred, estimate their probabilities, and find the most probable ones as a function of time, took 210 s. The discrete-time dynamic programming approach took 0.0056 s with the 0.1 s time step shown in the figure, but took 224 s with 10^4^ time steps. State sequence analysis for the HIV example took 50 s, whereas 1000 stochastic trajectories took 0.41 s (generation) plus 47 s (analysis), and discrete-time dynamic programming took 19 s with a one-day time step or 219 s with 10^4^ time steps. For the ion channel example, state sequences for each voltage level are solved separately. The amount of time for state sequence analysis at each voltage ranged from 5.6 s (at *V* = 0 mV) up to 26 s (at *V* = −100 mV), with an average time of 17 s. The dynamic programming approach, using the same 10^4^ time steps as state sequence analysis, takes 219 s per voltage with very little variability for different voltages. In these examples, then, state sequence analysis appears to be as efficient as or considerably more efficient than alternative approaches.

Given the significant and still-increasing interest in stochastic biochemical systems, we believe that state sequence analysis will find many other applications. The method can, in principle, be applied to arbitrary models expressed within the stochastic chemical kinetics formalism [22]. For some systems, maximum probability state sequences may be of only limited interest. For example, if we consider a stochastic gene expression system, it is probably not of concern exactly how the system might change from having *N*_1_ protein molecules at one time to *N*_2_ molecules at another time. However, we expect that maximum probability state sequences would be of interest in a number of other scenarios, such as studying how proteins fold, how protein complexes assemble or how different sequences of transcription factor binding events lead to the induction or repression of a gene. Continuous-time Markov chains are a central modelling formalism in many domains besides stochastic chemistry, such as queueing theory, fault diagnosis, reliability engineering, user modelling, etc. Thus, we believe that the mathematical problem we have formulated, of finding maximum probability sequences of state transitions, and our method of solving that problem constitute valuable new tools for analysing discrete-state continuous-time stochastic systems.

## 4. Material and methods

### 4.1. Computing state sequence probabilities

Suppose we are given a continuous-time Markov chain 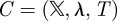. For any 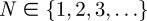 and any sequence of *N* + 1 states, 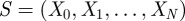, the time-dependent probability of the state sequence, 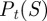, is defined as the probability that the chain would visit precisely that sequence of states by time *t*, given that it starts in state *X*_0_ at time zero. More formally, letting 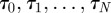 be dwell times in the states 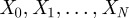, we can define4.1
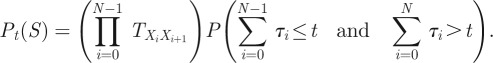
This is not, however, a computationally convenient definition, because of the second, time-dependent term on the right-hand side.

Given the state sequence *S*, we can obtain 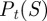, viewed as a function of time *t* over some interval 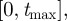 by analysing a different, but related, continuous-time Markov chain. Define a new chain 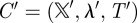 whose states correspond to possible sequences of states in chain *C*. Whenever chain *C*′ is in state *S*′, corresponding to state sequence 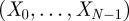 of chain *C*, let it dwell there for a random amount of time that is exponentially distributed with parameter 
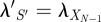
, and then transition randomly to a new state 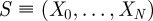 with probability 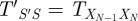. By construction, the probability that chain *C* transits the sequence *S* by time *t* is precisely the probability that chain *C*′ is in state *S* at time *t*.

Even if our original chain *C* has a finite state set, the derived chain *C*′ will in general have a countably infinite state set. Nevertheless, the state probabilities of the chain *C*′ obey the Chapman–Kolmogorov equations [16], namely4.2
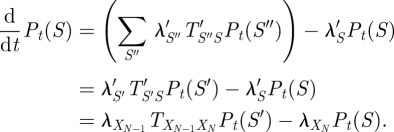
The first equation is just a statement of the Chapman–Kolmogorov equations for the chain *C*′. Intuitively, it says that: (1) probability mass flows into state *S* from possible predecessor states at a rate that depends on the predecessor's dwell time parameter and the transition probability from the predecessor to *S* and (2) probability mass flows out of state *S* at a rate proportional to the dwell time parameter of *S*. The second equality above derives from observing that state *S* has only one possible predecessor, namely *S*′, which corresponds to the one-step shorter sequence of states in the original chain *C*.

To obtain 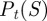, we need to merely solve the above linear differential equation. It is a non-homogeneous equation, because it depends on 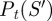, which varies with time. But of course, 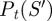 obeys its own linear differential equation, which depends in part on the probability of a state sequence that is yet one step shorter. Thus we can find 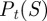 by solving the time-homogeneous system of linear differential equations—the Chapman–Kolmogorov equations—where the variables are 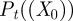, 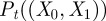, … , 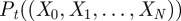. The initial conditions for the system are 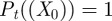 and 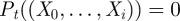 for 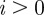. In some cases, the solution may have a simple analytical form. In other cases, one may prefer to obtain a numerical solution. In our Matlab and R implementations, available at http://www.perkinslab.ca, we have used built-in routines for numerical solution of differential equations to obtain the probabilities. By default, we ask the solvers to return the solutions on a time grid of 10^4^ steps spanning the time period of interest, though this can readily be changed. In any case, the number of equations in the system is *N* + 1, and solving these equations is relatively straightforward. Thus, we are able to obtain 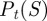 over the time interval of interest, 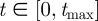.

### 4.2. Identifying the most probable state sequence using dominance and dynamic programming

Because there are, in general, infinitely many possible state sequences, we cannot simply evaluate all of their probabilities at some time of interest 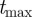 to find the maximally probable one. In order to guide the search for a maximally probable state sequence, we propose to use a notion we call *dominance*. Suppose *S*_1_ and *S*_2_ are two different state sequences that share the same initial and final states. We say that *S*_1_ dominates *S*_2_ if 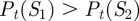 for all 
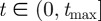
. If *S* is not dominated by any other sequence, then we say *S* is non-dominated. *S*_1_ and *S*_2_ cannot dominate each other if they start at different states or end at different states. Clearly, there is always a non-dominated sequence among 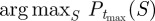. Otherwise, we could find a dominating sequence that would have an even higher value of 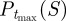. Moreover, we show in §4.4 that if the continuous-time Markov chain has a finite state set, 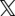, as it does in the examples in this paper, then there must be a finite number of non-dominated state sequences. If we can identify them all, and check them to see which has the largest probability, then we can guarantee finding the maximum probability sequence. The question, then, is how to enumerate non-dominated sequences.

A key observation is that if 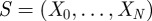 is non-dominated, then the one-step shorter sequence 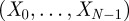 is also non-dominated. The sequences 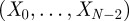, 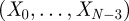 and so on, down to the sequence 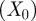 are also non-dominated. In other words, all prefixes of any non-dominated sequence must also be non-dominated. This is proved in §4.3. Assuming that the initial state of the system is known to be *X*_0_, we propose to enumerate non-dominated sequences from shorter to longer, starting from the trivial sequence 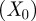. After enumerating the sequences, each is evaluated to see which is most probable at time 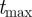. This is achieved by the following dynamic program.

- Initialize a list A to contain the sequence 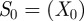.
- Initialize a list B to be empty.
- While list A is not empty:
  - Remove the first state sequence *S* from A.
  - Compute 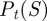 for 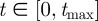.
  - If S is not dominated by any sequence on list B
    - Add *S* to list B.
    - Remove from B any sequences dominated by *S*, as well as any extensions of such sequences.
    - For each possible single-step extension of *S*, add the corresponding state sequence to the end of list A.
- Evaluate all sequences *S* on list B to find one maximizing 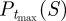.

Upon completion of the ‘while’ loop, all non-dominated sequences will be on list B. In the final step, these are evaluated for the one with maximum 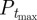. Using the earlier-mentioned basic idea, we can readily solve several other problem variants as well. For instance, if we also have an observed final state 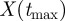, and we want to find the maximum probability state sequence that starts at 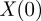 and ends at 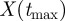, we need to merely restrict the maximization in the final step to be over sequences beginning and ending, respectively, at these two states. More generally, if we have an initial state probability distribution 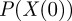 and a final state probability distribution 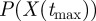, then we can find the most probable state sequence by employing two modifications. First, we must put all possible initial states, as singleton sequences, on the list A in the first step of the algorithm. Second, in the final step, we maximize the quantity 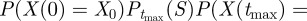
*X*_*N*_), where *X*_0_ and *X*_*N*_ are the first and last states in the sequence *S*, and 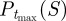 is the probability of the state sequence given the initial condition 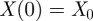. One can also compute not just the single most probable state sequence, but the *K* most probable sequences for 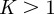. This can be done simply by changing the algorithm above to discard a sequence only if it is dominated by *K* others that have already been found. We use several of these variants in the HIV and ion channel examples.

### 4.3. Prefixes of non-dominated sequences are non-dominated

In the previous section, we claimed that prefixes of non-dominated sequences must also be non-dominated. Here, we prove this assertion. Recall from §4.1 that for any 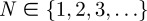, for a length-*N* state sequence 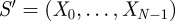 and any one-step extension of that sequence 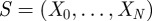, we have the Chapman–Kolmogorov relationship (equation (4.2)). The corresponding integral equation is4.3
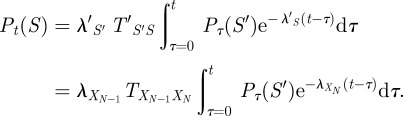
This can be confirmed by differentiating both sides of the equation. It also takes advantage of the fact that 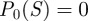 for any sequence other than 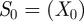. Otherwise, an additive constant would appear on the right-hand side. Now, let 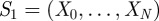 for 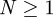 be a non-dominated state sequence, and let 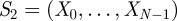 be the one step shorter sequence. Our assertion is that *S*_2_ is also non-dominated. To see this, assume the opposite—that there is a sequence 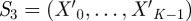 that dominates *S*_2_. This sequence may be of a different length than *S*_2_; that is, we allow 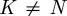. However, by the definition of dominance, this sequence must begin and end in the same states as *S*_2_. Hence, 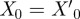 and 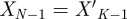. Let 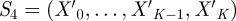 be the one-step extension of *S*_3_ that ends at the same state as *S*_1_, so that 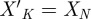. Then for any 
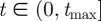
,
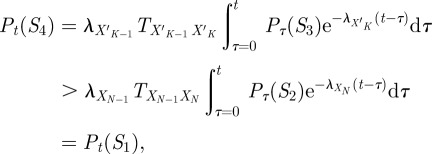
where the first and last steps come from equation (4.3) and the middle step comes from the assumption that *S*_3_ dominates *S*_2_. The conclusion 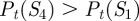, however, contradicts the assumption that *S*_1_ is non-dominated. Thus, *S*_2_ must be non-dominated.

### 4.4. Termination of the dynamic program for finite continuous-time Markov chains

In §4.2, we asserted that the number of non-dominated sequences is finite if the state set of the chain is finite. To be more precise, we mean that among the set of all possible sequences—those with non-zero probability—there is a finite number of non-dominated sequences. To see this, suppose that there is actually an infinite number of non-dominated sequences. Because there is a single start state, *X*_0_, and a finite number of possible final states for these sequences, there must be at least one possible final state, call it 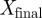, for which we have infinitely many non-dominated state sequences with non-zero probability. Let these sequences be denoted *S*_1_, *S*_2_, *S*_3_, … .

Now, recall that the probability 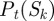 for 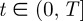 depends on three things: (i) the sequence of transitions must occur, (ii) the waiting times in all states except the last must finish by time *t*, and (iii) the chain must still be waiting in the last state at time *t*. We can upper-bound this probability by focusing just on the second condition. If 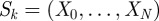, and if 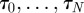 are the waiting times in these states, then4.4
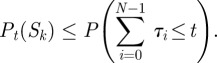
We can further upper-bound this probability by requiring only that each individual waiting time in all but the last state takes less than time *t*, so that4.5
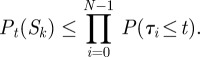
The different waiting times can have different waiting time parameters. However, suppose we let 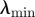 be the smallest waiting time parameter over all states in the chain, and let 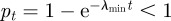 be the probability that a single such waiting time would finish in time *t*. Then4.6
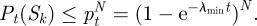
Because we have infinitely many sequences, *S*_1_, *S*_2_, *S*_3_, … , they must grow arbitrarily large in length. Let 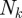 be the minimum number of transitions in any state sequence starting from the 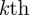 sequence in the list. Then4.7
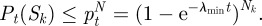
Given that 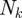 diverges to infinity with increasing *k*, we observe that 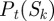 must converge pointwise to zero with increasing *k*. In particular, this means that 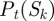 vanishes compared with 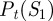 for all sufficiently large *k*; that is, *S*_1_ itself dominates all 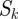 for sufficiently large *k*. This contradicts the assumption that we have infintely many non-dominated state sequences.

### 4.5. Estimating the HIV mutation model

For our HIV analysis, we used a dataset deposited in Genbank, a publicly available collection of DNA sequences, under accession nos. AY000001 to AY003708. The sequences contain 984 bp from the HIV-1 pol gene that were obtained from patients in phase II clinical studies (DMP 266-003, DMP 266-004 and DMP 266-005) of Efavirenz combination therapy. More about the dataset can be found in [34]. As Efavirenz is a reverse-transcriptase (RT) inhibitor, we focused only on the RT sections of the sequences. The final model was based on the mutations: L100I, K101Q, K101E, K103N, V106M, Y188L, Y188H, G190S, G190E, G190A and P225H.

We estimated a continuous-time Markov chain model from the data, using the 122 patients who had measurements at more than one time point. At some time points, multiple distinct HIV genotypes are present in a patient; we take the most common variant as representative of the state of the patient's HIV at that time. The states of our model are combinations of the selected mutations that occurred in the 122 patients. Twenty-two such states were identified; however, 3 of them were not observed to lead to or from any other states, and they were excluded from the model. The final model contains 19 states: wild-type, G190S, G190E, G190A, Y188L, Y188L + G190E, K103N, K103N + P225H, K103N + G190A, K103N + Y188H, 103N + V108I, K103N + V106M, K101E, K101E + G190S, K101Q, K101Q + G190S, K101Q + K103N, L100I + K103N and L100I + K103N + P225H. Thus, the model can be represented by a 19
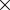
19 transition rate matrix.

Following previous analyses of the same dataset [40], we estimate the transition rates of our continuous-time Markov chain, using a method due to Albert [42]. The idea behind this approach is that if *q*(*i*,*j*) is the instantaneous transition rate from state *i* to state *j*, then it can be estimated as *N*(*i*,*j*)/*A*(*i*), where *N*(*i*,*j*) is the number of observed transitions from *i* into *j*, and 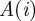 is the total time spent in state *i*. More precisely, suppose that at time 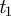 state *X*_1_ is observed in patient X, and at time 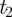, state *X*_2_ is observed. We make the simplifying assumption that the transition from *X*_1_ to *X*_2_ was a direct transition, and that the transition occurred at time 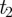 (hence, that pair of observations attributes 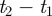 waiting time to state *X*_1_). Other assumptions on the transition times/rates are possible, but Foulkes & De Gruttola [38] found that alternative assumptions had little influence on the conclusions of their study; thus, we have not explored these alternatives. The estimated transition rates between states can then be separated into a dwell time parameter for the source state (for source state *i* it is 
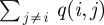
), and transition probabilities between states (for source state *i* and target state *j* it is 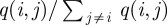). The resulting parameters are shown below.
state*λ* (unit of days)next states, probabilities in parentheseswild-type0.0061475G190S (0.044444) G190E (0.022222) G190A (0.011111)Y188L (0.044444) K103N (0.87778)G190S0.0082645wild-type (0.4) K101E + G190S (0.4) K101Q + G190S (0.2)G190E0G190A0.018182wild-type (1)Y188L0Y188L + G190E0.034483Y188L (1)K103N0.0038809wild-type (0.13043) K103N + P225H (0.3913)K103N + G190A (0.021739) K103N + Y188H (0.021739)103N + V108I (0.23913) K103N + V106M (0.021739)K101Q + K103N (0.086957) L100I + K103N (0.086957)K103N + P225H0.0015767K103N (1)K103N + G190A0K103N + Y188H0103N + V108I0.001005K103N (1)K103N + V106M0K101E0.014286wild-type (1)K101E + G190S0K101Q0.014085K101Q + K103N (1)K101Q + G190S0K101Q + K103N0.00088417K103N (1)L100I + K103N0.0027422K103N (0.66667) L100I + K103N +P225H (0.33333)L100I + K103N + P225H0

Probabilities of different state sequences are computed by solving the Chapman–Kolmogorov equations (equation (4.2)) using the ode45 function of Matlab. Matlab and R codes implementing the model and the state sequence analysis approach are posted on the website www.perkinslab.ca.

### 4.6. Details of the ion channel model and computations

The continuous-time Markov chain model of ion channel dynamics proposed by Vandenberg & Bezanilla [53] describes the voltage-dependent activation and latency of neural sodium conductances. The diagram in figure 3*a* shows the state transition diagram, with labels *a*, *b*, *c*, *d*, *f*, *g*, *i*, and *j* denoting transition rates between states. Each transition rate is the product of the dwell time parameter of the source state and the transition probability between the states. These voltage-dependent rates have the following generic form:4.8
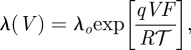
where *V* is the applied clamp voltage, 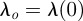 is the zero-voltage rate and *q* is the ‘effective valence’ of the transition. Also, *F* is the Faraday constant, *R* is the gas constant, and *T* is the absolute temperature. Throughout, 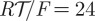, at 5°C. The numerical values for the effective valences and zero voltage rates for all but two of the possible transitions are listed below. (See figure 3 to match letters *a*, *b*, *c*, *d*, *f*, *g* to transitions between states.)
transition*q* (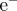)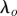 (s^−1^)*a*0.132969*b*−0.70704*c*1.2728932*d*−0.60725*f*0.49705*g*0.661117

The rates out of the inactivated state are 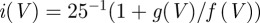 and 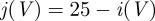. In order to compute the most probable state sequence during a closed interval of duration *T*, we cannot simply compute the most probable path starting and ending at *O*. Such a path may not even enter one of the closed states, let alone spend the specified amount of time among the closed states. At the start of a closed interval, there are two possible start states, *C*_3_ and *I*. These have initial probabilities proportional to the transition rate from *O* to each of these states, thus 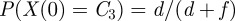 and 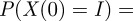
*f*/(*d* + *f*). Because we do not want any path to enter the open state until exactly time 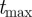, we alter the usual enumeration of possible state sequences so that only sequences that remain among the closed states are considered. Finally, for any non-dominated sequence ending at state *C*_3_ or *I*, we additionally multiply its probability by the likelihood of an instantaneous transition to state *O* at exactly time 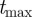. By definition, this likelihood is either the rate *c* or *j* for sequences ending at state *C*_3_ or *I*, respectively. This, then, forms the total ‘score’ for a path, which is optimized over all non-dominated paths that exclude transitions to state *O*. Matlab and R codes implementing the model and the state sequence analysis approach are posted on the website www.perkinslab.ca.

### 4.7. Time discretization and computation of maximum probability discrete-time trajectories

Given a continuous-time Markov chain 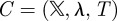 and a time step *Δ*, the standard discrete-time Markov chain approximation is given by 
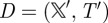
, where 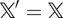 is the state set of the chain, and the transition probabilities *T*′ are determined as
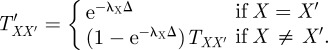
Having produced a discrete-time Markov chain from a continuous-time chain, we are then interested in computing maximum probability trajectories. For a discrete time chain, a trajectory to time *t* = *iΔ* simply specifies the state of the chain at each time 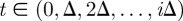. To compute maximum probability trajectories, we first assume a final time of interest, 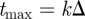. Define 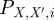 be the probability of the most probable path from state *X* to state 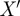 using precisely *i* steps (i.e. using total time 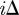). Let 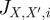 be the most probable path (or one of the most probable paths, in case several paths all have the same, maximum probability). We can compute *P* and *J* by a straightforward, well-known dynamic program [19]. We initialize as
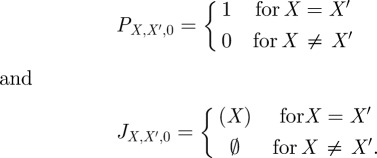
Then, we do the following:

- For *i* = 1 to *k*
  - For each 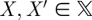    - Let 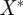 be any element of arg max_*X*″_ 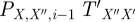    - Set 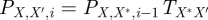    - Set 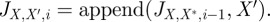

Here, append(*S*,*X*) adds state *X* to the end of sequence *S*. Upon completion, *J* holds a maximum probability path between any two states over any period of time up to 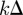.

## Footnotes

1 We note that there are alternative ways of constructing a discrete-time chain to approximate a continuous-time chain. The method suggested here, and described more generally in §4.7, is perhaps the simplest and has the advantage of not altering the structure of possible transitions between states—an important consideration if one's intention is to analyse possible system paths.

2 Although well known, this dynamic program has no name; see [19] or §4.7 for details. The algorithm can also be viewed as a special case of the Viterbi algorithm, with non-informative observations during the trajectory.
